# Development of the Nurses’ Occupational Stressor Scale

**DOI:** 10.3390/ijerph17020649

**Published:** 2020-01-19

**Authors:** Yi-Chuan Chen, Yue-Liang Leon Guo, Li-Chan Lin, Yu-Ju Lee, Pei-Yi Hu, Jiune-Jye Ho, Judith Shu-Chu Shiao

**Affiliations:** 1 School of Nursing, College of Medicine, National Taiwan University (NTU), No. 1, Ren-Ai Rd. Sec. 1, Taipei 10051, Taiwan; d05426004@ntu.edu.tw (Y.-C.C.); r00426019@ntu.edu.tw (Y.-J.L.); 2 Department of Environmental and Occupational Medicine, College of Medicine, National Taiwan University (NTU), No. 1, Ren-Ai Rd. Sec. 1, Taipei 10051, Taiwan; leonguo@ntu.edu.tw; 3 Department of Environmental and Occupational Medicine, National Taiwan University Hospital (NTUH), No. 7, Chung-Shan South Rd., Taipei 10002, Taiwan; 4 Institute of Clinical Nursing, National Yang-Ming University, No. 155, Sec. 2, Linong Street, Taipei 11221, Taiwan; lichan@ym.edu.tw; 5 Institute of Labor, Occupational Safety and Health (ILOSH), Ministry of Labor, No. 99, Lane 407, Hengke Rd., New Taipei City 22143, Taiwan; peiyi@mail.ilosh.gov.tw (P.-Y.H.); hjj@mail.ilosh.gov.tw (J.-J.H.); 6 Department of Nursing, National Taiwan University Hospital (NTUH), No. 7, Chung-Shan South Rd., Taipei 10002, Taiwan; 7 Occupational Health Nursing and Education Association of Taiwan (OHNEAT), No. 1, Ren-Ai Rd. Sec. 1, Taipei 10051, Taiwan

**Keywords:** nurse, occupational stressor, scale development, stressor scale, work environment

## Abstract

Although nurses work in stressful environments, stressors in such environments have yet to be clearly assessed. This study aimed to develop a Nurses’ Occupational Stressor Scale (NOSS) with high reliability and validity. Candidate questions for the NOSS were generated by expert consensus following focus group feedback, and were used to survey in 2013. A shorter version was then developed after examination for validity and reproducibility in 2014. The accuracy of the short version of the NOSS for predicting nurses’ stress levels was evaluated based on receiver operating characteristic curves to compare existing instruments for measuring stress outcomes, namely personal burnout, client-related burnout, job dissatisfaction, and intention to leave. Examination for validity and reproducibility yielded a shorter version of NOSS with only 21 items was considered sufficient for measuring stressors in nurses’ work environments. Nine subscales were included: (1) work demands, (2) work–family conflict, (3) insufficient support from coworkers or caregivers, (4) workplace violence and bullying, (5) organizational issues, (6) occupational hazards, (7) difficulty taking leave, (8) powerlessness, and (9) unmet basic physiological needs. The 21-item NOSS proved to have high concurrent and construct validity. The correlation coefficients of the subscales for test-retest reliability ranged from 0.71 to 0.83. The internal consistency (Cronbach’s α) coefficients ranged from 0.35 to 0.77. The NOSS exhibited accurate prediction of personal burnout, client-related burnout, job dissatisfaction, and intention to leave.

## 1. Introduction

As highlighted by the International Labour Organization [1], occupational stress is an increasingly global phenomenon which affects workers in all workplaces and countries. Nurses are known to have high work demands, high occupational stress, high rates of burnout [2,3,4], low job satisfaction [5], to experience workplace bulling [6], and may have mental health problems [7]. Job stress and burnout result from the cumulative effects of stressors in nursing work, and may consequently influence patient outcomes [8,9,10] and nurses’ intention to leave their jobs [11,12]. In accordance with the statistics of Taiwanese National Union of Nurses Associations (TUNA), presently only about 60% of licensed nurses in Taiwan practice [13]. However, Singapore and Australia have around 86.1% and 98.5% in active practice, respectively [14,15]. TUNA found 57.28% nurses had intention to leave nursing profession, and the three major reasons were about “salary and bonus”, “heavy workload” and “work–life imbalance”. Ref. [16] In the study of 10 European countries [17] showed 27.1% nurses considered to leave the workplace, and their perceptions of nursing practice environment was an important factor of retention.

In addition to applying stress management interventions to reduce stress [18], as recommended by Happell et al. [19], an initial step toward reducing occupational stress is to understand the stressors present in nurses’ work environments as well as the methods through which these stressors may be eliminated. Not only qualitative researches have been performed to look for nursing stressors [19,20], but several self-report scales have been developed to measure nursing stress indicators in hospital environment, such as Expanded Nursing Stress Scale (ENSS) [21], and Practice Environment Scale of the Nursing Work Index [22]. However, stressors vary widely in different cultures and are highly influenced by health care systems. Examples of variations include those in frequencies of on-call duty, patient-to-nurse ratios, reliance on patients’ families for daily partial care, regulated break times, and monetary compensation for overtime work. Additionally, although scales for measuring nursing stressors have been developed, measurements of psychological stress among nurses, including burnout, job dissatisfaction, and intention to leave, are rarely reported. The study developed a Nurses’ Occupational Stressor Scale (NOSS) to identify comprehensive nursing stressors. The scale was evaluated for validity and reliability and to examine relationships among stress indicators.

## 2. Materials and Methods

### 2.1. Study Design

Two cross-sectional studies were conducted to develop an instrument to measure nurses’ occupational stressors. This study was divided into three phases: (1) generation and pilot testing of candidate questions, (2) condensation of the scale according to validity and reproducibility, and (3) examination of accuracy of the condensed NOSS for predicting stress outcomes.

### 2.2. Participants and Ethical Considerations

In 2013, candidate questions for the NOSS were pilot tested on nurses who worked in hospitals with “excellent” ratings under the New Hospital Accreditation of 2012 in Taiwan. The condensed NOSS was tested in 2014. Participants were recruited from the population based on conformity and excellence under the New Hospital Accreditation between 2010 and 2013. The research protocols were approved by Research Ethics Committee of National Taiwan University Hospital with the approval numbers of 20130807RINC and 201407075RINA. Exemption of written consent was approved, and returned questionnaires were regarded as nurses’ willingness to participate in the study. No ethical issues occurred during the study period.

### 2.3. Data Collection

Stratified random sampling for the questionnaire surveys in 2013 and 2014 was conducted. Electable hospitals were sampled in proportion by hierarchy. The hospital managers were invited to approve the study via phone call. Nurses were recruited from multiple wards, namely the internal medicine ward, surgical ward, maternity and pediatric ward, intensive care unit, operating room, emergency department, psychiatric department, and outpatient department. The questionnaires were mailed to the participating hospitals and delivered to nurses. All returned questionnaires were previewed by the researchers and then recorded through optical mark reading.

The questionnaires were self-administered. The participants’ demographic characteristics, work environment traits, levels of personal burnout, client-related burnout, job satisfaction, and intentions to leave were inquired.

#### 2.3.1. Personal Burnout and Client-Related Burnout

A Chinese version of the Copenhagen Burnout Inventory (C-CBI) was developed with high internal consistency, constructive validity, and criterion-related validity [23]. Personal burnout and client-related burnout are two subscales in the C-CBI, containing five and six items, respectively, to assess the frequencies of specific scenarios within the preceding week on a 5-point Likert scale (0 to 4 representing “never” to “always”). Following Chin et al. [24], the cut-off point for nurses in the high burnout group was set as the 90th percentile.

#### 2.3.2. Job Dissatisfaction and Intention to Leave

Job dissatisfaction was assessed by the answer “somewhat unsatisfied” or “very unsatisfied” to the question, “Generally speaking, are you satisfied with your job?” Intention to leave a nursing job was assessed by the following three items: (1) Answering “unlikely” or “uncertain” to the question, “Do you intend to remain in your job for at least 2 more years?” (2) Obtaining a score of 7 or higher on the item, “Please rate your intention to leave on a scale of 0 to 10, with 0 being no intention to leave and 10 being highly considering leaving.” (3) Answering “once a month” or “more frequently” to the question, “How often do you think about leaving your job?”

### 2.4. Data Analysis

Data analysis was performed using JMP statistical software version 10.0 (SAS Institute, Cary, NC, USA). Descriptive statistics were calculated to summarize demographic characteristics. Test-retest reliability and internal consistency were examined by analyzing test-retest correlations and Cronbach’s α scores. Content validity was assessed by experts. Construct validity was calculated through common factor analysis. The suitability of factor analysis was inspected using the Kaiser–Meyer–Olkin (KMO) test [25] and Bartlett’s test of sphericity [26]. For all analyses, *p* < 0.05 was considered statistically significant.

## 3. Results

### 3.1. Phase 1: Generation and Pilot Testing of Candidate Questions

#### 3.1.1. Methods of Constructing Questions

The procedure for developing the NOSS is described sequentially as follows: information collection, content confirmation, format design, pretesting, panel discussion, expert validation, pilot study, and content determination.

After the literature review, the content of the NOSS was constructed according to research goals, and by referring to the questionnaire titled “survey of perceptions of safety and health in the work environment in 2013 Taiwan” [27] and the work–family conflict scale [28]. The researchers had the pretest to find unsuitable wordings and expert panel discussions for suggestions and clarification. The expert panel was composed of three professionals in the fields of nursing, psychiatry, and occupational medicine and six nurses from primary, secondary, and tertiary hospitals.

#### 3.1.2. Content Validity Index

Expert validity was assessed after revision of the panel discussion. The experts were the aforementioned three professionals on the panel. NOSS items were scored on a Likert-type scale (1 and 2: modification required; 3: related; 4: strongly related). After alteration or deletion of inadequate items (scores lower than or equal to 2), the content validity index of the NOSS was 0.81.

#### 3.1.3. Phase 1 Questionnaire Survey

A total of 72 hospitals rated “excellent” under the 2012 Hospital Accreditation were our target hospitals. Of these 72 hospitals, 13 were tertiary hospitals, 41 were secondary hospitals, and 18 were primary hospitals. In 2013, Stratified random sampling and questionnaire survey of 7 tertiary, 10 secondary, and 2 primary hospitals was conducted. A total of 2956 questionnaires were issued and 2796 were returned. After exclusion of men, nurse managers, nurse practitioners, and incomplete questionnaires, 1781 questionnaires were deemed eligible for analysis. The effective response rate was 60.3%.

The participants’ demographic characteristics are summarized in Table 1; their mean age was 30.3 years, most were single (64.3%), and most had an educational level of college or above (63.1%). The average total working tenure was 8.6 years.

The internal consistency scores assessed by Cronbach’s α were 0.92 for personal burnout and 0.90 for client-related burnout. The mean scores for personal burnout and client-related burnout were 63.9 and 47.9, respectively. Of the participants, 15.5% harbored intentions to leave their jobs.

#### 3.1.4. Construct Validity

The KMO score (0.93) and Bartlett scores (chi-square statistic = 51,378.93; degrees of freedom = 990; *p* < 0.001) indicated that factor analysis may be practical. Common factor analysis was performed to assess the construct validity of the NOSS, resulting in 10 factors with eigenvalues greater than 1.0 (Table 2). Relying on the assumption that the dimensions of the scale were correlated or uncorrelated, we implemented Varimax (orthogonal rotation) and Promax (oblique rotation) both. The outcomes showed that Varimax and Promax grouped the same items into 10 factors. The cumulative variability of these extracted 10 factors explained by varimax was 51.8%.

#### 3.1.5. Test-Retest Reliability

A convenience sample of 50 hospital nurses from northern, central, and southern Taiwan was invited to assess test-retest reliability; 36 pairs of test-retest questionnaires were completed. Test-retest reliability scores were calculated through Pearson’s correlation with a 2-week interval. The Pearson’s correlation coefficients of the 10 subscales were 0.75, 0.72, 0.74, 0.75, 0.72, 0.75, 0.71, 0.76, 0.72, and 0.61. The test-retest reliability of the whole NOSS was 0.84.

#### 3.1.6. Internal Consistency Reliability

Most NOSS items were scored on a 4-point Likert scale (1 to 4 representing “strongly disagree” to “strongly agree”), whereas 5 items were reverse scored. The average total score of the NOSS was 107.1 (SD = 14.2), ranging from 65 to 158. A higher score indicated a higher frequency of work stressors experienced by the participant in question. Cronbach’s α was used to measure the internal consistency. The internal consistency scores of the 10 NOSS subscales were 0.88, 0.92, 0.87, 0.86, 0.35, 0.63, 0.86, 0.78, 0.06, and 0.63. The internal consistency of the whole NOSS was 0.89.

### 3.2. Phase 2: Condensation of the NOSS According to Validity and Reproducibility

The initial NOSS underwent a condensation process to reduce item numbers. All items on the condensed NOSS were selected from the original NOSS. Items were examined as independent variables, and personal burnout, client-related burnout, job dissatisfaction, and intention to leave were set as dependent variables. The selection algorithms were based on predictions of dependent variables and reliability. Items with favorable prediction were prioritized for inclusion in the condensed scale and those without favorable prediction or low reliability were re-examined through panel discussions.

First, common factor analysis of the 43 items selected for the condensed scale yielded 10 factors (Table 2): “work demands”, “work–family conflict”, “insufficient support from coworkers or caregivers”, “workplace violence and bullying”, “organizational issues”, “occupational hazards”, “difficulty taking leave”, “powerlessness”, “interpersonal relationships”, and “unmet basic physiological needs”. Since the item numbers differed among factors, the total score of each factor was adjusted to between 0 and 100. Table 3 presents the areas under the receiver operating characteristic curves (AUCs) used to examine sensitivity and specificity. Forward stepwise with a *p* value of 0.1 was implemented to examine predictions of indicators under each of the 10 factors. Factors 1, 2, 4, 6, and 7 were significantly related to personal burnout (AUC = 0.79). Factors 1, 2, 3, 4, 7, and 8 were significantly related to client-related burnout (AUC = 0.80). Factors 1, 2, 6, 7, and 10 were significantly related to job dissatisfaction (AUC = 0.75). Factors 1, 2, 4, 5, 7, and 10 were significantly related to intention to leave (AUC = 0.75). Factor 9—interpersonal relationships—was not significantly related to any indicators.

The stability of the NOSS was assessed through evaluation of test-retest reliability. The values of the 10 factors ranged from 0.61 to 0.76. After setting the minimum stability value of 0.70 [29], factor 10—with a stability value of 0.61—was revised.

A range of 0.3–0.7 was set for internal consistency reliability by recommendation [30]. The internal consistency reliability of the NOSS factors ranged from 0.35 to 0.92, except for factor 9 (0.06). Prominent items were preserved to represent the concept of each factor. Because of the conceptual similarity between factor 9 (interpersonal relationships) and factor 3 (insufficient support from coworkers or caregivers), two items of factor 9 (“I am worried that the incompetence of my colleagues will affect patient safety” and “The primary caregivers do not execute their tasks appropriately”) were reclassified under factor 3 and all other items under factor 9 were omitted.

### 3.3. Phase 3: Examination of Accuracy of the Condensed NOSS for Predicting Stress Outcomes

#### 3.3.1. Phase 3 Questionnaire Survey

After revision, the participants for the confirmation survey were sampled from 417 hospitals in 2014. A total of 71 candidate hospitals (1 tertiary, 7 secondary, and 63 primary hospitals) were sampled. A total of 3974 nurses were recruited, and 3786 returned the questionnaires. Under the same exclusion criteria as those of the 2013 survey, 2655 questionnaires were deemed eligible for analysis, yielding an effective response rate of 66.8%. The participants’ demographic characteristics are shown in Table 1.

#### 3.3.2. Test-Retest Reliability of the 21-Item Condensed NOSS

Of 50 nurses who worked in primary, secondary, and tertiary hospitals in Taiwan, 48 completed the test-retest study within one week. According to Pearson’s correlation, the p values of the nine factors ranged from 0.71 to 0.83. The test-retest reliability of the overall 21-item NOSS was 0.76.

#### 3.3.3. Internal Consistency Reliability of the 21-Item Condensed NOSS

Table 4 presents the item-to-subscale correlations. The Cronbach’s α scores of the subscales ranged from 0.35 to 0.77 except for “workplace violence and bullying”, which contained only one item, and thus lacked internal consistency reliability. The internal consistency of the 21-item NOSS as a whole was 0.91.

#### 3.3.4. Comparison of the prediction accuracy of the original NOSS and condensed NOSS

To examine predictions of intermediate markers by the original and condensed NOSSs, the two scales were compared with respect to personal burnout, client-related burnout, job dissatisfaction, and intention to leave (Table 5). The AUCs for the indicators ranged from 0.73 to 0.82 on the condensed NOSS. Among the participants of the first year survey, the AUCs for the original 43-question version ranged from 0.75 to 0.80 and those for the condensed version ranged from 0.75 to 0.81. These results suggested that the condensed NOSS might be equally sensitive and specific to the original NOSS for predicting nurses’ stress outcomes.

**Figure 1:**
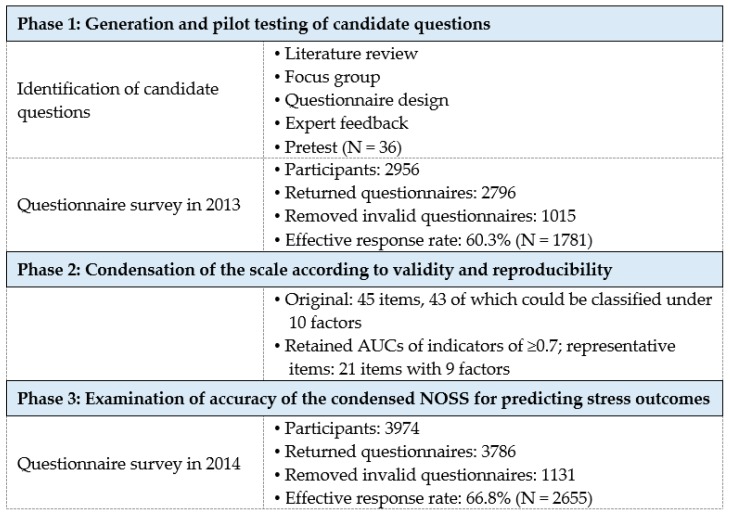
The process of the NOSS development.

The process of the NOSS development is illustrated in Figure 1.

## 4. Discussion

This study constituted the effort to develop a stressor scale for nurses in Asia.Despite measurements for stress reactions being used extensively, workplace factors, namely stressors among hospital nurses, are rarely characterized or quantified. The ENSS contains 57 items and was tested on 2280 randomly selected nurses; the scale was found to be correlated with overall life stress items and health problem indices [21]. The NOSS has three major advantages: (1) comprehensive assessment of nursing work traits that could interfere with life, including occupational hazards, workplace violence and bullying, difficulty taking leave, and unmet basic physiological needs; (2) 21 items only, so relatively little time required for completion; and (3) comparisons with four important stress indicators in both surveys and reasonable prediction of these outcomes.

This section discusses the results of using factors of the NOSS and indicators for confirmation. Burnout is regarded as a response to job stressors correlated with excessive direct contact with patients [3,31]. Hence, “work demands”, “insufficient support from coworkers or caregivers”, and “workplace violence and bullying” [6,12] may be reasonable factors for predicting personal burnout and client-related burnout. Work–life conflict was regarded as a strong predictor of burnout [32,33]. Consequently, the relationship between “work–family conflict” and burnout is predictable. According to the World Health Organization [34], ergonomic hazards is one of potential health hazards among health care workers. Studies conducted in Hong Kong and Japan have revealed that manually lifting patients or heavy objects is a risk factor associated with musculoskeletal disorders such as lower back pain [35,36]. Other researchers observed that lower back pain was related to personal burnout [37]. Thus, it seems plausible that “occupational hazards” factor is associated with personal burnout. Taking a sick leave or a leave for family-related reasons is not easy for Japanese nurses; without substitutes, other nurses need to work harder or more hours to compensate. Thus, nurses may feel guilty about taking leaves, and inability to take leaves could lead to burnout or even overwork death [38]. The “difficulty taking leave” factor may reliably predict burnout. Items under the “powerlessness” factor have been verified as being associated with client-related burnout. Due to higher frequency of suffering patients contact, nurses might experience greater compassion fatigue than other professionals [3]. Burnout can easily occur among those caring for dying people [39], and the associated feeling of powerless and the inability to deliver effective care to such people could cause moral dilemmas and burnout [40].

Researchers observed a negative relationship between job satisfaction and nursing tasks left undone [41]. Furthermore, nursing care may be forced out of a work schedule by non-nursing tasks, and neglected nursing care was found to be a strong predictor of intention to leave [42]. These findings may match the relationships of the “work demands” factor with job dissatisfaction and intention to leave in the study.

Confrontations with patients and their families may be another nursing stressor [43]; however, the relationships of this item with job dissatisfaction and intention to leave were nonsignificant. A study among physicians revealed that job satisfaction decreased and intention to leave increased when “work–family conflict” increased [44]. Besides, nurses were dissatisfied with inadequate protective equipment when caring for highly infectious patients [45]. This may support our finding of a relationship between “occupational hazards” and job dissatisfaction. In a meta-analysis [46], availability and use of work–family support policies positively related to job satisfaction and intention to stay. In short, the “difficulty taking leave” factor may reliably predict burnout, job dissatisfaction, and intention to leave. For decades, nurses’ meal breaks and rest breaks have been regarded as a factor possibly related to job satisfaction and intention to stay [47]. Instances of nurses holding their urine or decreasing their water consumption were recorded [48]. Accordingly, the items categorized under the “unmet basic physiological needs” factor may be common in Taiwan and China.

One item on the NOSS is rather culturally unique; despite patients’ family members not being intuitively recognized as having such collegial relationships as those that nurses have with patients, family members have long made commitments to care for hospitalized patients because of the Chinese value of filial piety [49]. Because hospitals reduce nursing manpower to minimize costs, a portion of care depends on family members or private attendants. Therefore, unsurprisingly, “feeling stressed because primary caregivers do not execute their tasks appropriately” predicts client-related burnout.

“I have to maintain professional units other than my own” was found to be related to personal burnout, client-related burnout, job dissatisfaction, and intention to leave. These outcomes were observed when nursing units had temporary shortages of personnel. However, one would imagine the nurses who worked in another unit could face unfamiliar medical equipment, coworkers and an unfamiliar environment. These likely induce additional stress.

This study had some limitations. First, males were excluded. The distribution of male nurse in our study was 2.4% (n = 46) and that in Taiwan was approximately 1.6% at the time of the study [13]. Because the exclusion of male nurses had no impact on the results (data not shown), only female nurses were analyzed. Second, questionnaires with any missing item were excluded to ensure the accuracy of developing NOSS, which was the major reason of effective response rates less than 70%. There was not significant different of participants’ demographics between valid and invalid questionnaires (data not shown). Third, the NOSS was developed for hospital nurses; thus, the scale might not be applicable to clinics or nursing homes. Fourth, nurses unable to adapt to given work environments, had left the profession, or had transferred to less stressful environments were not included. Therefore, a healthy worker effect or healthy worker survival effect [50] may be present, and this could have led to underestimation of stress in the study. Fifth, although a single factor is suggested to include at least three items [51], we decided not to ignore less items to detect nursing stressors due to uniqueness of nursing clinical environment. Accordingly, factor 4 had only one item and factor 6, factor 7, factor 8, and factor 9 had only two items. The AUCs of the first- and second-year observations revealed that the 21-item NOSS may be adequate for predicting indicators of common stress among nurses. Finally, our questionnaire did not contain items about participant’s income, economic burden or job insecurity. As described in previous studies, global economic crisis could have caused hospital budgets reduction, and consequently led to medical supply shortage, increased workload and job insecurity [52,53]. Thus, economic crisis was regarded as an important stressor related to workers’ mental health status [52]. Further studies may consider financial factors while assess nursing practice environment and related outcomes.

The strengths of the study are described as follows. First, this study analyzed a nationally representative sample based on stratified sampling. Second, in both surveys, rather large numbers of nurses completed the questionnaire, enabling examination of factors and their relationships with stress indicators. Third, the identified stressors in this study were individually related to the subscales of burnout, job dissatisfaction, and intention to leave. The identified stressors can be applied in other countries if pretesting for comparisons with stress indicators is conducted.

## 5. Conclusions

This current study developed NOSS, which identified nine groups of occupational stressors in nursing practice environments, as well as predicting personal burnout, client-related burnout, job dissatisfaction, and intention to leave. Using this scale, stressors in nurses’ work environment can be measured, and while intervention is applied, the effectiveness of such intervention can be evaluated.

## Figures and Tables

**Table 1:** Participant characteristics.

Variable	Eligible Participants in 1st Year (*N* = 1781)				Eligible Participants in 2nd Year (*N* = 2655)			
	*n*	%	Mean	SD	*n*	%	Mean	SD
Age (years)			30.3	6.6			32.5	7.3
≤30	1022	57.4			1152	43.4		
31–40	619	34.8			1097	41.3		
≥40	135	7.6			365	13.7		
Missing	5	0.3			41	1.5		
Marital status								
Single	1145	64.3			1439	54.2		
Married	606	34.0			1145	43.1		
Divorce or widow	27	1.5			53	2.0		
Missing	3	0.2			18	0.7		
Educational level								
Professional school	20	1.1			93	3.5		
Junior college	634	35.6			921	34.7		
College or above	1124	63.1			1631	61.4		
Missing	3	0.2			10	0.4		
Total work tenure (years)			8.6	6.8			10.1	7.3
<5	729	40.9			877	33.0		
5–10	508	28.5			694	26.1		
11–15	259	14.5			516	19.4		
16–20	183	10.3			310	11.7		
≥20	101	5.7			236	8.9		
Missing	1	0.1			22	0.8		
Working hours/day			9.3	1.1			9.2	1.2
Working hours/week			49.9	8.1			47.8	7.7
Sleeping hours/day			6.8	1.3			6.8	1.2
Personal burnout (standardized score: 0–100)			63.9	21.0			59.9	20.5
Personal burnout ≥ 95	230	12.9			241	9.1		
Client-related Burnout (standardized score: 0–100)			47.9	18.7			46.8	19.8
Client-related burnout ≥ 75	190	10.7			290	10.9		
Score of intention to leave (score: 0–10)			5.6	2.8			5.4	2.7
Had intention to leave	255	15.5			255	10.4		

**Table 2:** Factor loadings for items loaded on 10 factors of the 43-item Nurses’ Occupational Stressor Scale (NOSS) through varimax rotation.

Subscales and Component Items	Factor Loading
**1. Work Demands**	
I am worried about receiving complaints from patients or their relatives for not meeting their demands.	0.68
I have to bear the negative sentiment of patients or their relatives.	0.76
I do not have sufficient time to meet patients’ and their relatives’ demands.	0.74
I am unsure of the extent of patients’ conditions or treatments that I should reveal to them.	0.59
Excessive duties in the workplace prevent me from attending to patients.	0.61
I have to maintain professional units other than my own.	0.49
**2. Work–Family Conflict**	
The burden of work affects my domestic life.	0.75
The amount of time my job occupies makes it difficult for me to fulfill family responsibilities.	0.83
The burden of work makes it difficult for me to undertake my personal chores and/or engage in hobbies.	0.83
My job produces strain that makes it difficult for me to fulfill my family duties.	0.83
I have to adapt my schedule for family activities/outings to accommodate my work responsibilities.	0.74
**3. Insufficient Support from Coworkers or Caregivers**	
The professional evaluation of care of mine is opposite to that of the doctors.	0.62
Doctors’ temperamental nature agitates me.	0.56
I cannot instantaneously obtain patient-related information because of inadequate communication within the team.	0.68
Team members do not appear to help in a timely manner under urgent circumstances.	0.65
Lack of support from the team affects patients’ trust in me.	0.63
**4. Workplace Violence and Bullying**	
Verbal abuse such as insults and sarcastic comments.	0.66
Psychological abuse such as threats, discrimination, bullying, and harassment.	0.83
Physical abuse such as hitting, kicking, pushing, pinching, pulling, and dragging.	0.71
Sexual harassment such as inappropriate implications and behaviors.	0.68
**5. Organizational Issues**	
The on-call system affects my life.	0.66
My working hours include on-call hours.	−0.48
I was informed of a change to my schedule at less than 24 hours’ notice.	0.54
The organization usually remunerates my overtime work at a low rate of pay.	0.40
Not achieving a promotion (e.g., level 1 or 2) within the expected period affects my income.	0.45
**6. Occupational Hazards**	
Exposure to chemicals such as chemotherapy drugs, alcohol, and Cidex.	0.63
Exposure to radiation or strong light such as X-ray, ultraviolet light, and lasers.	0.67
I feel stressed considering that my patients might be have contagious diseases such as SARS or AIDS ^a^.	0.38
Transporting patients or equipment.	0.61
The workplace offers sufficient protective equipment such as masks and gowns.	−0.35
**7. Difficulty Taking Leave**	
The level of difficulty in asking for leaves for household emergencies is_______% (0% = very difficult, 100% = very smooth) ^b^.	0.87
The level of difficulty in excusing myself for feeling strong discomfort is_______% (0% = very difficult, 100% = very smooth) ^b^.	0.80
**8. Powerlessness**	
Patients’ conditions do not improve.	0.66
Encountering the death of a patient.	0.70
I have insufficient time to offer mental health care to patients during working hours.	0.45
**9. Interpersonal Relationships**	
Relationships among colleagues within the unit are generally good.	−0.34
I should teach student nurses and newcomers while caring for patients.	0.55
I worry that my colleagues’ incompetence will affect patient safety.	0.45
The manager or head nurse supports me in the event of a conflict between me and a patient.	−0.38
Primary caregivers do not execute their tasks appropriately.	0.38
**10. Unmet Basic Physiological Needs**	
I have no time to fulfill my personal needs (e.g., water consumption and toilet breaks).	0.61
I cannot take an uninterrupted 30-minute mealtime break.	0.80
I can receive deserved compensation such as premiums and compensatory leave for overtime of more than 1 h.	−0.47

Note: Factor loadings of >0.32 are recognized on the subscale. ^a^ SARS: severe acute respiratory syndrome; AIDS: acquired immune deficiency syndrome. ^b^ Transfer of percentages to scores are explained as follows: 0%–25% = 4, 26%–50% = 3, 51%–75% = 2, 76%–100% = 1. Items not classified under any factors in the table: “I cannot complete my duties or required tasks during working hours” and “In the preceding month, I have used _______ hour(s) of my free time to handle documents from the hospital for accreditation or unit-related affairs”.

**Table 3:** Personal burnout, client-related burnout, job dissatisfaction, and intention to leave as indicators for item retention on the NOSS (N = 1781).

Factor	Personal Burnout ^a^		Client-Related Burnout ^b^		Job Dissatisfaction ^c^		Intention to Leave ^d^	
	OR	AUC	OR	AUC	OR	AUC	OR	AUC
1	1.04 ***	0.68	1.05 ***	0.73	1.06 ***	0.77	1.06 ***	0.78
2	1.04 ***	0.68	1.06 ***	0.75	1.04 ***	0.69	1.04 ***	0.67
3	1.03 ***	0.64	1.04 ***	0.70	1.05 ***	0.71	1.05 ***	0.73
4	1.02 ***	0.64	1.03 ***	0.70	1.03 ***	0.70	1.04 ***	0.71
5	1.02 ***	0.64	1.02 ***	0.64	1.02 ***	0.63	1.02 ***	0.63
6	1.02 ***	0.60	1.03 ***	0.69	1.03 ***	0.67	1.03 ***	0.68
7	1.02 ***	0.66	1.01 ***	0.62	1.01 ***	0.63	1.01 ***	0.61
8	1.02 ***	0.59	1.03 ***	0.64	1.02 ***	0.61	1.02 ***	0.62
9	1.02 ***	0.63	1.04 ***	0.69	1.04 ***	0.68	1.04 ***	0.70
10	1.00	0.51	0.99	0.53	0.99	0.53	0.99 *	0.53

*Note*: OR: odds ratio; AUC: areas under the receiver operating characteristic curves. * *p* < 0.05, *** *p* < 0.001. ^a^ The standardized total score for personal burnout was ≥ 65. ^b^ The standardized total score for client-related burnout was ≥ 95. ^c^ “Somewhat unsatisfied” and “Quite unsatisfied” were classified as job dissatisfaction. ^d^ Intention to leave was defined as “unlikely to or uncertain about staying in the job for another two years”, “score on the scale of leaving the job ≥ 7”, and “thinking about leaving once in a month or more frequently”.

**Table 4:** Statistics of the 21-item NOSS (*N* = 2655).

Item		Mean	SD	Cronbach’s α if the Item is Deleted
*Subscale 1: Work Demands* (Cronbach’s α: 0.61)				
1	I have to bear negative sentiment from patients or their relatives.	3.16	0.66	0.44
2	Excessive duties in the workplace prevent me from attending to patients.	3.01	0.73	0.58
3	I have to maintain professional units other than my own.	3.24	0.72	0.50
*Subscale 2: Work–Family Conflict* (Cronbach’s α: 0.70)				
4	The burden of work affects my domestic life.	2.89	0.69	0.79
5	The burden of work makes it difficult for me to undertake my personal chores and/or engage in hobbies.	2.82	0.73	0.53
6	I have to adapt my schedule for family activities/outings to accommodate my work responsibilities.	3.11	0.65	0.52
*Subscale 3: Insufficient Support from Coworkers or Caregivers* (Cronbach’s α: 0.62)				
7	Doctors’ temperamental nature agitates me.	3.14	0.69	0.48
8	I worry that my colleagues’ incompetence will affect patient safety.	2.85	0.68	0.56
9	I feel stressed because primary caregivers do not execute their tasks appropriately.	2.96	0.65	0.53
*Subscale 4: Workplace Violence and Bullying*				
10	I feel stressed due to psychological abuse such as threats, discrimination, bullying, and harassment.	2.85	0.76	-
*Subscale 5: Organizational Issues* (Cronbach’s α: 0.59)				
11	The on-call system affects my life.	2.97	0.82	0.45
12	The organization usually remunerates my overtime work at a low rate of pay.	2.77	0.81	0.47
13	Not achieving a promotion (e.g., level 1 or 2) within the expected period affects my income.	3.04	0.76	0.55
*Subscale 6: Occupational Hazards* (Cronbach’s α: 0.39)				
14	I feel stressed considering that my patients might be have contagious diseases such as SARS or AIDS.	3.21	0.65	-
15	I need to transport patients or equipment.	3.12	0.76	-
*Subscale 7: Difficulty Taking Leave* (Cronbach’s α: 0.77)				
16	I cannot ask for leaves for household emergencies.	2.87	0.83	-
17	I cannot excuse myself for feeling strong discomfort.	2.60	0.84	-
*Subscale 8: Powerlessness* (Cronbach’s α: 0.35)				
18	It upsets me if patients’ conditions do not improve.	2.77	0.65	-
19	I have insufficient time to offer mental health care to patients during working hours.	3.00	0.67	-
*Subscale 9: Unmet Basic Physiological Needs* (Cronbach’s α: 0.69)				
20	I have no time to fulfill my personal needs (e.g., water consumption and toilet breaks).	2.81	0.75	-
21	I cannot take an uninterrupted 30-minute mealtime break.	3.02	0.81	-

**Table 5:** Revision and confirmation of the NOSS.

Variable	Personal Burnout			Client-Related Burnout			Job Dissatisfaction			Intention to Leave		
	Factors	AUC	R^2^	Factors	AUC	R^2^	Factors	AUC	R^2^	Factors	AUC	R^2^
43-item NOSS in 1st year (*N* = 1781)	1 2 4 6 7	0.79	18.81%	1 2 3 4 7 8	0.80	18.52%	1 2 6 7 10	0.75	14.41%	1 2 4 5 7 10	0.75	12.16%
21-item NOSS in 2nd year (*N* = 2655)	1 2 4 6 7	0.82	20.64%	1 2 3 4 7 8	0.77	15.67%	1 2 6 7 9 ^a^	0.73	11.20%	1 2 4 5 7 9 ^a^	0.77	13.93%
21-item NOSS in 1st year (*N* = 1781)		0.81	19.93%		0.79	19.13%		0.76	14.73%		0.75	12.87%

^a^ What was originally factor 9 was deleted and factor 10 became the new factor 9 in the 21-item NOSS.
